# Genome Sequence and Analysis of the Flavinogenic Yeast *Candida membranifaciens* IST 626

**DOI:** 10.3390/jof8030254

**Published:** 2022-03-01

**Authors:** Margarida Palma, Stephen Mondo, Mariana Pereira, Érica Vieira, Igor V. Grigoriev, Isabel Sá-Correia

**Affiliations:** 1 Institute for Bioengineering and Biosciences, Instituto Superior Técnico, Universidade de Lisboa, Av. Rovisco Pais, 1049-001 Lisboa, Portugal; mariana.r.pereira@tecnico.ulisboa.pt (M.P.); erica.vieira@tecnico.ulisboa.pt (É.V.); isacorreia@tecnico.ulisboa.pt (I.S.-C.); 2 Department of Bioengineering, Instituto Superior Técnico, Universidade de Lisboa, Av. Rovisco Pais, 1049-001 Lisboa, Portugal; 3 Associate Laboratory i4HB—Institute for Health and Bioeconomy at Instituto Superior Técnico, Universidade de Lisboa, Av. Rovisco Pais, 1049-001 Lisboa, Portugal; 4 US Department of Energy Joint Genome Institute, Lawrence Berkeley National Laboratory, Berkeley, CA 94720, USA; sjmondo@lbl.gov (S.M.); ivgrigoriev@lbl.gov (I.V.G.); 5 Department of Agricultural Biology, Colorado State University, Fort Collins, CO 80523, USA; 6 Department of Plant and Microbial Biology, University of California Berkeley, Berkeley, CA 94720, USA

**Keywords:** *Candida membranifaciens*, Debaryomycetaceae, yeast isolation, soil, riboflavin, flavinogenic yeasts, iron metabolism, xylose assimilation

## Abstract

The ascomycetous yeast *Candida membranifaciens* has been isolated from diverse habitats, including humans, insects, and environmental sources, exhibiting a remarkable ability to use different carbon sources that include pentoses, melibiose, and inulin. In this study, we isolated four *C. membranifaciens* strains from soil and investigated their potential to overproduce riboflavin. *C. membranifaciens* IST 626 was found to produce the highest concentrations of riboflavin. The volumetric production of this vitamin was higher when *C. membranifaciens* IST 626 cells were cultured in a commercial medium without iron and when xylose was the available carbon source compared to the same basal medium with glucose. Supplementation of the growth medium with 2 g/L glycine favored the metabolization of xylose, leading to biomass increase and consequent enhancement of riboflavin volumetric production that reached 120 mg/L after 216 h of cultivation. To gain new insights into the molecular basis of riboflavin production and carbon source utilization in this species, the first annotated genome sequence of *C. membranifaciens* is reported in this article, as well as the result of a comparative genomic analysis with other relevant yeast species. A total of 5619 genes were predicted to be present in *C. membranifaciens* IST 626 genome sequence (11.5 Mbp). Among them are genes involved in riboflavin biosynthesis, iron homeostasis, and sugar uptake and metabolism. This work put forward *C. membranifaciens* IST 626 as a riboflavin overproducer and provides valuable molecular data for future development of superior producing strains capable of using the wide range of carbon sources, which is a characteristic trait of the species.

## 1. Introduction

*Candida membranifaciens* is an anamorphic yeast first described as *Candida melibiosi* var. *membranifaciens* by Lodder & Kreger-van Rij [1]. Based on the examination of strains assigned to *Candida melibiosi* var. *membranifaciens*, *C. melibiosi* var. *melibiosi*, and *C. guilliermondii*, Wickerham and Burton observed that the variety *membranifaciens* was unable to mate and able to ferment melibiose more vigorously than *C. melibiosi* [2]. *Candida melibiosi* var. *membranifaciens* was proposed as the new species *C. membranifaciens*. More recently, upon phylogenetic analysis of the D1/D2 domains of the large subunit and the nearly complete small subunit rRNA genes from species that express the coenzyme Q-9 form, *C. membranifaciens* was phylogenetically placed in the *Yamadazyma* clade of Debaryomycetaceae family [3]. Currently, this species is taxonomically allocated to the *Yamadazyma*/*Candida* clade. 

*Candida membranifaciens* strains have been isolated from diverse habitats, including fresh, marine, and estuarine waters, insects, plants, and clinical specimens [4,5]. This species has the ability to assimilate a wide variety of carbon sources that include hexoses (glucose and galactose), pentoses (xylose and arabinose), α-glucosides (maltose, trehalose, melezitose), β-glucosides (cellobiose, salicin), β-fructosides (sucrose, inulin), and α-galactosides (raffinose, melibiose) [4]. It is also able to grow on media with high osmolarity [4], consistent with its presence in marine environments. Another interesting physiological attribute was found in one strain isolated from seawater, *Candida membranifaciens* subsp. *flavinogenie* W14-3, described as a flavinogenic yeast [6]. Regardless of all those remarkable features, its biotechnological potential is poorly explored. For instance, riboflavin production by other *C. membranifaciens* strains has not been studied. 

Riboflavin is a precursor of the coenzymes flavin mononucleotide (FMN) and flavin adenine dinucleotide (FAD), being required for biochemical reactions in all living cells [7,8]. It is produced by plants and by the majority of the microorganisms, but humans and animals are unable to synthesize this vitamin, which makes riboflavin an essential component of their diet [9]. In 2012, the annual production of riboflavin worldwide was 9000 t, of which about 70% were used as animal feeding additive and the remaining 30% as a food additive and in pharmaceutical applications [10]. Although, in industry, riboflavin can be produced by chemical or biological synthesis, the bio-based synthesis has replaced chemical synthesis due to economic and environmental reasons [11]. The bioprocesses that dominate riboflavin production use the Gram-positive bacterium *Bacillus subtilis* and the hemiascomycete filamentous fungi *Ashbya gossypii* [10]. The industrial production of riboflavin by the yeast *Candida flareri* (formerly *Candida famata*, teleomorph *Debaryomyces subglobosus*) has also been considered, but it was interrupted due to the genetic instability of industrial strains [12,13]. Other flavinogenic yeast species include *Meyerozyma guilliermondii* (formerly, *Pichia guilliermondii*), *C. albicans*, *C. parapsilosis*, *C. tropicalis*, *Schwanniomyces occidentalis*, *Debaryomyces hansenii, and C. membranifaciens* subsp. *flavinogenie* [6,12,13,14,15]. These species are also members of the Debaryomycetaceae family and belong to the CTG clade, where CTG codon is predominantly translated as a serine instead of a leucine [16]. Another interesting observation is that these flavinogenic yeasts overproduce riboflavin only under iron-limiting conditions [17]. The influence of iron availability in flavinogenesis has long been a topic of debate [6,12,18], but the mechanisms underlying riboflavin overproduction in response to iron depletion are still poorly understood.

Microorganisms are considered overproducers of riboflavin when they accumulate over 10 mg/L of this vitamin [12]. Based on the degree of riboflavin accumulation, microorganisms can fall into three categories: weak (10–100 mg/L), moderate (up to 600 mg/L), and strong (more than 1 g/L) overproducers [15]. Moderate overproducers are, for example, the species mentioned above from the genera *Candida*, *Debaryomyces*, and *Meyerozyma* that can accumulate the referred riboflavin concentrations upon culture media optimization. Strong overproducers, such as *B. subtilis*, *A. gossypii*, and *C. flareri*, can accumulate more than 10 g/L riboflavin [19,20,21]. Overproducing strains have been developed and selected mainly using random or site-directed mutagenesis or by overexpressing genes involved in riboflavin biosynthesis and these genetic manipulations were combined with culture medium optimization [11]. *C. membranifaciens* subsp. *flavinogenie* W14-3 is a weak overproducer; after the optimization of culture conditions, this strain was able to accumulate 22 mg/L of riboflavin in the growth medium [22]. This value is remarkably below the 20 g/L reported to be produced by a mutagenized, although unstable, *C. flareri* strain [21]. Nevertheless, there is still room for the selection and development of new overproducer yeasts, with potential for stable riboflavin production from raw materials other than defined media. Yeasts can offer some technological advantages over filamentous fungi, including low growth requirements, easier distribution of the cells along the fermenter, and the possible use of yeast biomass as a source of protein [23]. Therefore, the identification of novel riboflavin yeast overproducers and their physiological and molecular characterization are essential steps to gain new insights into relevant unclear issues. This is the case of the regulation of riboflavin secretion and accumulation in the culture medium, the characterization of the unknown phosphatase that catalyzes the dephosphorylation of 5-amino-6-ribitylamino-2,4(1H,3H) pyrimidinedione 5′-phosphate in riboflavin biosynthetic pathway, and the physiological role of riboflavin overproduction under iron-limiting conditions. Moreover, from a circular bioeconomy perspective, the selection of new yeast strains able to effectively use organic residues as alternative feedstocks for the production of added-value compounds such as riboflavin is a topic to be pursued. 

In this work, we isolated four *C. membranifaciens* strains from soil and investigated their potential to overproduce riboflavin and *C. membranifaciens* IST 626 was found to produce the highest amounts of riboflavin. Riboflavin production by this strain was optimized by manipulating growth medium composition. The genome of *C. membranifaciens* IST 626 was sequenced and annotated. A comparative genomic analysis with other relevant yeast species is also provided.

## 2. Materials and Methods

### 2.1. Isolation and Identification of Candida membranifaciens Isolates

*Candida membranifaciens* isolates were obtained from three soil samples collected in Arrábida Natural Park, Sesimbra, Portugal (38°26′12.4′′ N, 9°04′05.0′′ W); Berlengas Biosphere Reserve, Berlenga Grande, Portugal (39°24′59.0′′ N, 9°30′23.7′′W); and Ferreira do Alentejo, Portugal (38°02′43.6′′ N, 8°06′34.3′′ W). For yeast isolation, three cycles of culture enrichment were performed to avoid growth of filamentous fungi, as previously described [24]. Approximately one gram of each soil sample was inoculated in 50 mL of growth medium containing: 3 g/L malt extract (Sigma-Aldrich, Burlington, MA, USA), 3 g/L yeast extract (ThermoFisher), 5 g/L peptone (ThermoFisher), 1 g/L (NH_4_)_2_SO_4_ (Panreac), 0.25 g/L KH_2_PO_4_ (Panreac, pH 5.0), 30 g/L of glucose (Scharlau), and 30 g/L of xylose (Sigma-Aldrich). This growth medium with the soil sample was supplemented with chloramphenicol (100 µg/mL) and incubated at 30 °C at 150 rpm for 48 h (First Enrichment). Then, 1 mL of this culture was added to 49 mL of the same medium, and incubated again at 30 °C, 150 rpm, 48 h (Second Enrichment). To differentiate yeasts with the ability to grow in different carbon sources, a differential enrichment step was performed, where 1 mL from the Second Enrichment culture was added to 49 mL of the same medium but containing either 60 g/L glucose or 60 g/L xylose and incubated in the same conditions as before. After 48 h of cultivation, the samples were diluted in 0.85% NaCl solution and poured into isolation agar medium. This medium includes 3 g/L yeast extract (ThermoFisher), 5 g/L peptone (ThermoFisher), 1 g/L (NH_4_)_2_SO_4_ (Panreac), 0.25 g/L KH_2_PO_4_ (Panreac), and 20 g/L agar (NZYtech), with either 60 g/L glucose or 60 g/L xylose [24], and was supplemented with chloramphenicol (100 µg/mL). Plates were incubated at 30 °C for 48 h. Yeast cells from colonies with different morphologies were observed on an Axioplan microscope (×1000 magnification) (Zeiss^®^) and streaked into new agar plates to assure the purity of the isolate. Yeast isolates were maintained at 4 °C until DNA extraction was performed. For long-term storage, isolates were preserved at −80 °C in their isolation medium containing 15% (*v/v*) glycerol. 

For the molecular identification of yeast isolates, genomic DNA was extracted using the phenol:chlorophorm:isoamyl alcohol method [25] and used as a template for the amplification by polymerase chain reaction (PCR) of the D1/D2 domain sequence of the 26S and the internal transcribed spacer (ITS) region of ribosomal DNA (rDNA). The primers’ pairs NL-1 (5’-GCATATCAATAAGCGGAGGAAAAG-3’) and NL-4 (5’-GGTCCGTGTTTCAAGACGG-3’), and ITS1 (5’-TCCGTAGGTGAACCTGCGG-3’) and ITS4 (5’-TCCTCCGCTTATTGATATGC-3’), known to be effective for the taxonomic identification of yeasts [26], were used in the amplification of D1/D2 and ITS regions, respectively. The two DNA fragments from each isolate were purified using NZYGel pure (NZYtech, Portugal) and Sanger-sequenced (Stabvida, Portugal) using each corresponding primer. The molecular taxonomic identification was performed by comparing D1/D2 and ITS sequences with others deposited in GenBank using the BLAST algorithm from the National Center for Biotechnology Information (NCBI) (http://www.ncbi.nlm.nih.gov/blast, accessed on 30 October 2019). The consensus sequences from D1/D2 region of *C. membranifaciens* strains IST 495, IST 498, IST 507, and IST 626 were deposited in GenBank under the accession numbers MZ614941, MW003712, MW003715, and MW532700, respectively. The consensus sequences from ITS region of *C. membranifaciens* strains IST 495, IST 498, IST 507, and IST 626 were deposited under the accession numbers MZ615411, MW003718, MW003721, and MW532702, respectively.

### 2.2. Phylogenetic Analysis

For the phylogenetic placement of *C. membranifaciens* strains isolated in this study, their D1/D2 domain of LSU rDNA sequence were aligned iteratively with the sequences of related species from Debaryomycetaceae family, retrieved from the GenBank under the accession numbers indicated in the phylogenetic tree, by using the multiple alignment tool Muscle [27]. The software MEGA-X v.10.2.2 was used for phylogenetic tree construction using the maximum likelihood method of the Tamura-Nei evolutionary model [28]. The confidence level of the clades was estimated using bootstrap analysis with 1000 replicates.

### 2.3. Yeast Strains Tested and Growth Media and Conditions Used

*Candida membranifaciens* strains IST 495, IST 498, IST 507, and IST 626, isolated and identified as described herein, were examined in this study. We also used *C. membranifaciens* PYCC 2525^T^ obtained from the Portuguese Yeast Culture Collection (PYCC). The basal growth media used in this study were prepared with either 6.9 g/L of commercial yeast nitrogen base without amino acids (mentioned hereafter as YNB) or 6.9 g/L yeast nitrogen base without amino acids and without iron (mentioned hereafter as YNB-Fe), both from Formedium^TM^ (United Kingdom), supplemented with 20 g/L glucose (Merck) or 20 g/L xylose (Sigma-Aldrich). All media and solutions were prepared using Milli-Q^®^ water (Merck Millipore) and all growth assays were carried out in 50 mL shake flasks containing 25 mL of medium. Pre-cultivation of yeast cells was performed in either YNB or YNB-Fe with 20 g/L glucose for 24 h at 30 °C with orbital shaking (250 rpm). After pre-cultivation, yeast cells were harvested by centrifugation (5000 *g*, 5 min) and inoculated at an initial optical density (OD) at 600nm of 0.1 ± 0.05 in 25 mL of the medium to be tested. To investigate the ability of different *C. membranifaciens* strains to produce riboflavin, yeast cells were inoculated in either YNB or YNB-Fe with 20 g/L glucose for 120 h under the growth conditions described above. To evaluate the impact of iron supplementation in the production of riboflavin, *C. membranifaciens* IST 626 cells were inoculated in YNB-Fe with 20 g/L of glucose, to which increasing concentrations of iron (III) chloride (+0.5 µM, +0.8 µM, +1.0 µM, +1.2 µM, +1.5 µM, +2.0 µM) were added. Riboflavin, biomass (OD_600nm_) and glucose concentrations were determined after 120 h of growth. Cells inoculated in YNB with 20 g/L of glucose were used as a control. To evaluate the impact of different sugars and glycine supplementation of the growth medium on riboflavin production by *C. membranifaciens* IST 626, cells were cultivated in YNB-Fe media containing either 20 g/L glucose or 20 g/L xylose as a carbon source and supplemented with 1 g/L or 2 g/L glycine (Sigma-Aldrich). 

### 2.4. Quantification of Riboflavin

Extracellular riboflavin was determined using a spectrophotometric method as previously described [29,30], but with minor modifications. Briefly, a volume of 1 mL of each culture was harvested and centrifuged at 10,000 rpm for 3 min to remove cells. Supernatants were mixed in 0.1 N HCl using the appropriate dilution, and riboflavin concentrations were determined by reading the absorbance of each sample at 445 nm in a spectrophotometer (Hitachi U-2001) and the riboflavin calibration curve performed using several dilutions of a pure riboflavin (Sigma-Aldrich) stock solution (0.2 g/L). 

### 2.5. Genome Sequencing, Assembly, and Annotation

Genomic DNA from *C. membranifaciens* IST 626 was sequenced in an Illumina Novaseq 6000 platform, producing 2 × 150-bp paired-end reads. Library preparation (NEBNext^®^ DNA Library Prep Kit from NewEngland Biolabs, Inc., Ipswich, MA, USA) and sequencing were carried out by Novogene Bioinformatics Technology Co., Ltd. (Hong Kong). Illumina sequencing produced 21,753,322 raw paired-end reads. Low-quality bases and adapters were removed using BBDuk from BBMap package (http://jgi.doe.gov/data-and-tools/bb-tools/, accessed on 30 June 2020). Read duplicates were removed using PRINSEQ (v0.20.4) (6). Ultimately, 17,196,026 high-quality reads were used for subsequent analysis. Correction of the reads and assembly into scaffolds were performed using SPAdes (v3.14.0) (7). Scaffolds smaller than 2000 bp were filtered out, and the remaining sets of scaffolds were used as draft assemblies. Assembly quality was analyzed using Quality Assessment Tool for Genome Assemblies (QUAST; v4.6.3) (8). The final assembly consists of 56 contigs, with a total length of 11,508,125 bases, which includes a mitochondrial DNA contig with 28,672 bp. The sequencing reads were deposited in Sequence Read Archive (SRA) under the accession number PRJNA777779, and the sequences obtained in the genome assembly were deposited in GenBank under the accession number JAKQXL000000000. During submission to NCBI, three scaffolds were determined to be contaminants and were excluded. The genome was annotated using the JGI Annotation pipeline [31], made available in JGI fungal genome portal MycoCosm (https://mycocosm.jgi.doe.gov/Canmem1, accessed on 25 February 2022).

### 2.6. Analysis of Genome Content

Gene prediction was performed by using the tools available at MycoCosm, the JGI’s web-based fungal genomics resource, at the JGI portal [31]. The functional analysis of predicted genes was based on the eukaryotic orthologous groups of proteins (KOG) classification [31,32]. Proteins involved in riboflavin biosynthesis and transport, as well as the proteins involved in iron homeostasis and transport were analyzed using the Annotation and Blast analysis tools from JGI. Members of sugar porter family (2.A.1.1) present in the genome of *C. membranifaciens* IST 626 were obtained based on JGI data for transporter annotations [31], which uses the Transport Classification (TC) system [33,34]. Glycoside hydrolases were obtained based on CAZy annotations [34] also available at MycoCosm.

### 2.7. Search for Putative Transcription Factor Binding Sites

Putative transcription factor (TF) binding sites were searched for in promoter regions of *C. membranifaciens* selected genes using YEASTRACT+ [35,36]. This database contains all known regulatory associations between transcription factors and target genes in different yeast species for which the information is available in the literature, which means, essentially, for *Saccharomyces cerevisiae.* YEASTRACT+ includes genomic information on the flavinogenic yeast species *C. albicans* [14]. The sequences of promoters of interest were retrieved from MycoCosm at the JGI portal [31,37] and inserted into the “Find TF Binding Site(s)” tool of YEASTRACT+ portal (http://yeastract-plus.org/pathoyeastract/calbicans, accessed on 30 June 2021). The analysis was performed using documented TF binding sites from *C. albicans*. 

## 3. Results and Discussion

### 3.1. Isolation and Identification of Candida membranifaciens Isolates

In this study, four different isolates were isolated from the superficial layer of three different soils, two of them from reserve areas in Arrábida Natural Park and Berlengas islands. For those isolations, a rich and undefined growth medium containing glucose and xylose was used for the first two enrichment steps and either one of these sugars was used in the third enrichment step [24]. Strains IST 626, IST 495, and IST 507 were isolated in a medium where xylose was the carbon source present in the third enrichment step, whereas strain IST 498 was isolated when glucose was the carbon source present in that enrichment step. 

**Figure 1:**
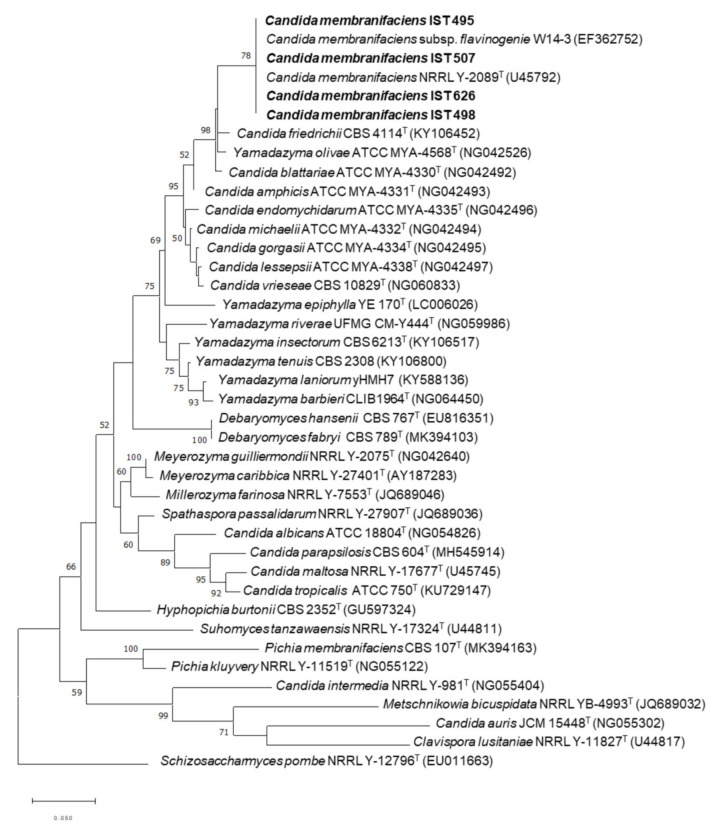
Phylogenetic placement of *C. membranifaciens* strains IST 495, IST 498, IST 507, and IST 626 isolated in this study. Phylogenetic analysis was based on the alignment of sequences of the D1/D2 domain of the 26S rDNA region, inferred by using the maximum likelihood method and Tamura–Nei model. Sequences from the type strains (superscript T) of different yeast species were included. GenBank accession numbers are shown in parentheses. The strains isolated in this study are highlighted in bold. The scale bar indicates the number of expected substitutions per site. The numbers provided on branches are frequencies with which a given branch appeared in 1000 bootstrap replications. The tree was rooted with *Schizosaccharomyces pombe*.

The isolates were identified based on the comparison of their D1/D2 and ITS sequences with the sequences deposited in the NCBI database. The sequences shared 100% identity with the corresponding *C. membranifaciens* sequences, and the isolates were considered of the *C. membranifaciens* species. The phylogenetic analysis, based on D1/D2 domain of LSU rDNA sequence, placed the isolates close to *C. membranifaciens* NRRL Y-2089^T^ (=PYCC 2525^T^) and the flavinogenic W14-3 strain on *Yamadazyma*/*Candida* clade (Figure 1). The closest species to the *C. membranifaciens* clade is *C. friedrichii* [3]. 

The natural habitat of *C. membranifaciens* is not defined, but isolates from this species have been retrieved from diverse habitats and substrates, including fresh, marine, and estuarine waters, insects, plants, and clinical specimens [4,5]. Yeasts of *Candida* genus have been isolated from soils worldwide [19,38,39,40], but, to the best of our knowledge, this is the first report on the isolation of the species *C. membranifaciens* from this habitat. Interestingly, in our study, four strains from this species were isolated from three different soils, showing that soil is a reservoir of this species. 

### 3.2. Candida membranifaciens Isolates Are Riboflavin Producers

**Figure 2:**
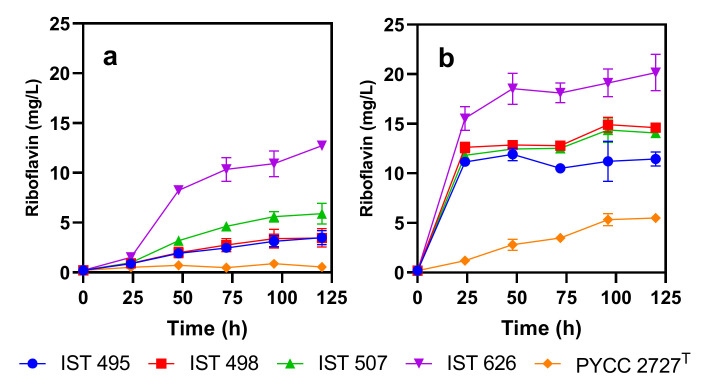
Riboflavin production by *C. membranifaciens* strains IST 495, IST498, IST 507, and IST 626 isolated in this study, and the type strain PYCC 2727^T^. All strains were cultured in YNB (**a**), or YNB-Fe (**b**). All media contained 20 g/L of glucose. Riboflavin production was determined during 120 h of growth at 30 °C and orbital agitation (250 rpm). Error bars represent the standard deviations of three independent measurements.

*C. membranifaciens* IST 626 was able to produce over 10 mg/L riboflavin when cultured in YNB with 20 g/L of glucose (Figure 2a). The other strains tested produced lower concentrations of the vitamin under the same conditions (Figure 2a). When the five strains examined were cultured in YNB-Fe, all strains except PYCC 2727^T^ overproduced riboflavin (produced more than 10 mg/L). *C. membranifaciens* IST 626 produced the highest concentration of this vitamin, which reached approximately 20 mg/L after 120 h of cultivation (Figure 2b). These results indicate that, contrary to *C. membranifaciens* PYCC 2727^T^, the four *C. membranifaciens* strains isolated in this study are flavinogenic. Moreover, the results confirm that the presence of iron has a marked negative impact on riboflavin production by *C. membranifaciens,* consistent with previous observations [6].

**Figure 3:**
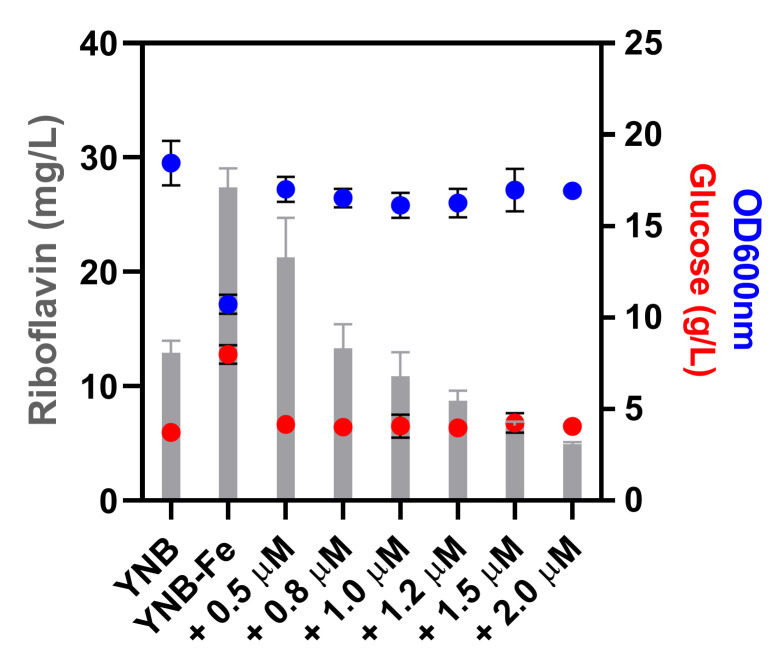
Riboflavin (bars), biomass (OD_600nm_) (blue circles), and glucose (red circles) concentrations determined after 120 h of cultivation of *C. membranifaciens* IST 626 in media containing different iron concentrations. *C. membranifaciens* IST 626 was grown under standardized conditions in YNB and in YNB-Fe to which increasing concentrations of iron (III) chloride (+0.5 µM, +0.8 µM, +1.0 µM, +1.2 µM, +1.5 µM, +2.0 µM) were added. All media contained 20 g/L of glucose. Riboflavin and glucose concentrations, and OD_600nm_ were determined after 120 h of growth at 30 °C and orbital agitation (250 rpm). Error bars represent the standard deviations of three independent measurements.

**Figure 4:**
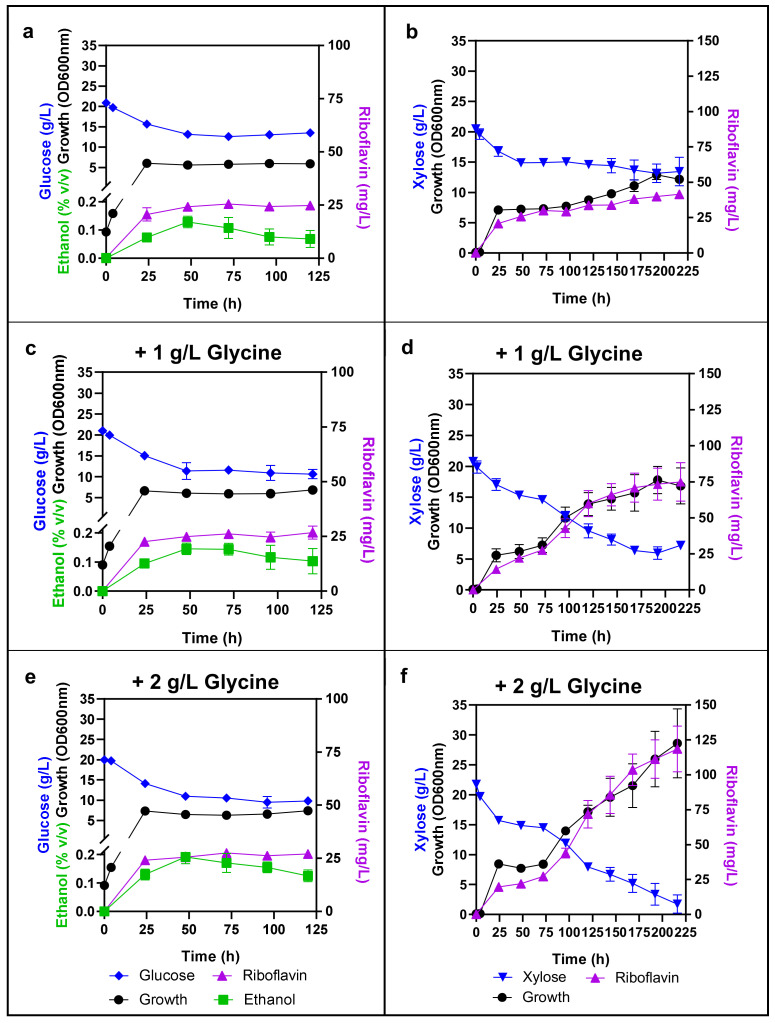
Glycine supplementation of the growth medium increases riboflavin volumetric production by *C. membranifaciens* IST 626 when xylose is the available carbon source. This strain was cultured in YNB-Fe supplemented with 20 g/L of either glucose (**a**,**c**,**e**) or xylose (**b**,**d**,**f**), without glycine (**a**,**b**), with 1 g/L of glycine (**c**,**d**), or 2 g/L of glycine (**e**,**f**). Samples were collected every 24 h in cells grown at 30 °C with an orbital agitation (250 rpm). Cultivations in media containing glucose were performed for 120 h and cultivations in media containing xylose were extended to 216 h. Results are the mean of three independent experiments.

Based on the higher riboflavin production capacity of *C. membranifaciens* IST 626, this strain was selected for further studies that included the determination of the effect of iron addition to YNB-Fe (Figure 3) and the optimization of growth medium conditions for riboflavin production (Figure 4). The supplementation of YNB-Fe with increasing concentrations of iron (III) chloride decreased riboflavin production by *C. membranifaciens* IST 626 in a dose-dependent manner (Figure 3). Among the conditions tested, the concentration of riboflavin produced and of remaining glucose was higher and biomass concentration was lower when *C. membranifaciens* IST 626 was cultured for 120 h in medium without iron. When increasing concentrations of iron (III) chloride were added to commercial YNB-Fe, a dose-dependent decrease in riboflavin concentration was observed after 120 h of growth. The concentration of cells and glucose was similar in all iron-(III)-chloride-supplemented media. The commercial medium YNB (containing ~1.2 µM iron (III) chloride) was used as a control. This study demonstrates the impact of different concentrations of iron (III) chloride in the volumetric production of riboflavin and shows that, in the absence of iron, biomass production and glucose consumption are negatively affected due to iron limitation. 

In the case of *C. membranifaciens* IST 626, riboflavin overproduction (>10 mg/L) was observed when iron (III) chloride was added at concentrations below 1.0 µM. The link between iron metabolism and flavinogenesis has long been established [15] but, after 40 years of research, the molecular and physiological mechanisms underlying such a relationship in flavinogenic yeasts remain poorly understood. Under iron depletion, riboflavin has been suggested to play a role in the nonenzymatic reduction of insoluble Fe^3+^ to the more accessible soluble Fe^2+^ or to act as a cofactor for the activity of intra- and extracellular enzymes [12]. This mechanism is well documented in some bacterial species [41,42], but was not validated in flavinogenic yeasts [12]. Iron is a vital micronutrient that is essential for multiple biological processes, including respiration, given that respiratory complexes contain heme and Fe–S clusters whose synthesis depends on iron availability [43]. In *S. cerevisiae*, at concentrations bellow 1 µM Fe, the cell faces iron deficiency and prioritizes the utilization of this micronutrient, meaning that most of the available iron goes to the mitochondria, where it is assembled into Fe–S clusters and heme centers, as well as prosthetic groups that are critical for cellular metabolism [44]. Other cellular processes can be severely affected by iron starvation due to the decrease in the availability of iron-dependent metabolites, such as amino acid intermediates, heme, unsaturated fatty acids, and deoxyribonucleotides [45], which may lead to a deceleration or even abrogation of several metabolic processes, for instance, sugar metabolization. The higher glucose concentration present in the medium without iron after 120 h of cultivation (Figure 3) might be the result of the deceleration of growth and sugar metabolism.

Comparison of riboflavin production by *C. membranifaciens* IST 626 cells cultured in either glucose (Figure 4a) or xylose (Figure 4b) showed that the volumetric production of this vitamin was higher when xylose was the available sugar, reaching approximately 33 mg/L after 120 h of cultivation. Ethanol was produced by *C. membranifaciens* IST 626 when glucose was the available sugar (Figure 4b,c,e). The percentage of glucose diverted to either alcoholic fermentation or respiration in this species has not been studied but, as in *S. cerevisiae*, *C. membranifaciens* was also able to produce ethanol from glucose under aerobic conditions. In contrast, xylose was assimilated but not fermented by this species (Figure 4b,d,f).

Based on the reported increase in riboflavin production by *C. flareri* (*C. famata*) and by *A. gossipy* when cultivated in media supplemented with glycine [21,46], the effect of glycine supplementation in YNB-Fe media containing either glucose or xylose was also assessed (Figure 4). Although glucose consumption and cell growth were slightly favored by glycine supplementation during the first two days of fermentation, riboflavin production by *C. membranifaciens* IST 626 was not enhanced by glycine addition to glucose YNB-Fe media (Figure 4a,c,e) and, for this reason, growth curves in these media were only followed for 120 h. Differently, riboflavin production was stimulated by the addition of glycine to the culture medium containing xylose (Figure 4b,d,f). Riboflavin concentration reached approximately 120 mg/L and xylose was almost fully consumed after 216 h of cultivation in medium supplemented with 2 g/L glycine (Figure 4f). The increase in riboflavin production in glycine-containing media correlates with the increase in yeast biomass, indicating that glycine was essential for the full catabolism of the xylose present. In the flavinogenic yeast *C. flareri*, the mechanisms underlying the enhancement of riboflavin production by glycine were not clarified [21]. Nevertheless, it is known that, in *S. cerevisiae*, glycine participates in multiple biological processes. They include, for example, the biosynthesis of purines, glyoxylate [47], of glutathione [48], and of serine by the glycine decarboxylase multienzyme complex that plays a critical role in connecting the metabolism of one-, two-, and three-carbon compounds in different metabolic pathways [49]. The positive impact of amino acids on the catabolism of other carbon sources by a different nonconventional yeast was previously demonstrated [50]. Although our results do not elucidate how glycine relates to catabolization of xylose and, consequently, increased biomass and riboflavin production, overall, they indicate that the production of this vitamin by *C. membranifaciens* remarkably increased upon optimization of the growth medium with xylose and glycine and that this strain can be further exploited to produce this vitamin.

Having in mind the future exploration of this strain for riboflavin production, it was considered of interest to obtain the first genome sequence for the species *C. membranifaciens* and the best riboflavin-producing strain IST 626 and provide its assembly and annotation. This is expected to contribute to enlightening the molecular mechanisms underlying riboflavin biosynthesis and the ability of this species to assimilate a wide variety of carbon sources. 

### 3.3. General features of Candida membranifaciens IST 626 Genome

The genome sequence of *C. membranifaciens* IST 626 was obtained by paired-end Illumina sequencing. A total of 17 million reads were acquired and assembled into 56 scaffolds (≥2000 bp), resulting in an overall sequence coverage of 224x. A summary of genome assembly statistics is presented in Table 1. 

**Figure 5:**
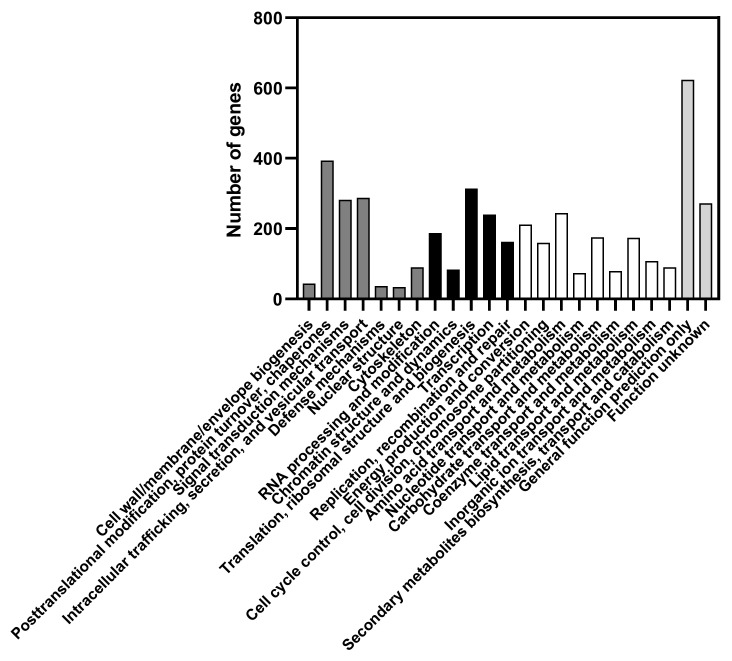
Number of predicted genes assigned to a function based on the eukaryotic orthologous groups of proteins (KOG) classification. Represented is the distribution of predicted genes according to their putative function within the major categories “Cellular processes and signaling” (dark grey), “Information storage and processing” (black), “Metabolism” (white), and “Poorly characterized functions” (light grey).

The sum of all scaffold sizes is 11,508,125 bp. The predicted GC content is 32.15%. This value is below the minimum value on the range of the GC content of other species from the *Candida*/*Yamadazyma* clade (GC content varies from 33.5 to 53.9%) [51,52,53]. A total of 5619 genes were predicted to be encoded in the genome of *C. membranifaciens* IST 626. Protein functions were assigned to 61% (3446 genes) of the predicted genes according to the eukaryotic orthologous groups of proteins (KOG) classification [54] (Figure 5). Among them, 1165 genes were assigned to “Cellular processes and signaling”, 980 genes to “Information storage and processing”, and 1301 genes to “Metabolism” major categories. In this latter category, the most dominant functions are “Amino acid transport and metabolism” (243 genes), “Energy production and conversion” (210 genes), “Carbohydrate transport and metabolism” (174 genes), and “Lipid transport and metabolism” (172 genes). A total of 893 genes (16% of the assigned genes) were included in the “Poorly characterized functions” category, which includes the categories “Function unknown” and “General function prediction only”. The number of genes assigned to each function is detailed in Supplementary file 1, where the number of genes assigned to other species related to *C. membranifaciens* is also included. 

### 3.4. Genomic Information and Corresponding Metabolic Traits 

#### 3.4.1. Proteins Associated with Riboflavin Production and Transport

Riboflavin biosynthesis comprises a total of seven enzymatic reactions controlled by six *RIB* genes and an unknown gene that codes for an enzyme catalyzing the dephosphorylation of 5-amino-6-ribitylamino-2,4(1H,3H) pyrimidinedione 5’-phosphate (ARPP). The phosphatase responsible for this reaction in *Arabidopsis thaliana* was recently described [55], but its homolog in yeast remains unknown. The genome of *C. membranifaciens* IST 626 contains the homologs of *RIB1*, *RIB2*, *RIB3*, *RIB4*, *RIB5*, and *RIB7* (Table 2), as well as the genes involved in the synthesis of flavin mononucleotide (FMN) (*FMN1*) and flavin adenine dinucleotide (FAD) (*FAD1*), which are essential cofactors for the majority of flavoproteins/flavo-coenzymes in different organisms [7,8]. To search for the missing phosphatase in the riboflavin biosynthetic pathway in *C. membranifaciens* IST 626 genome, the protein sequence of *A. thaliana* 5-amino-6-(5-phospho-D-ribitylamino)uracil phosphatase (AtPyrP2) [55] was used as a query in JGI’s MycoCosm Blast search tool. Two candidate proteins for the dephosphorylation of ARPP were identified (Table 2), which belong to the group of HAD hydrolyses, as in *A. thaliana*.

The overexpression of riboflavin biosynthetic genes in flavinogenic yeasts leads to the increase in riboflavin production in different species (reviewed in [57]). For instance, in *C. famata* the overexpression of the riboflavin biosynthetic genes *RIB1* and *RIB7* and of the transcriptional activator *SEF1* remarkably increased riboflavin production [58]. This transcription factor is essential for riboflavin production in *C. famata* and *P. guilliermondii* [12,59] and plays a role in iron homeostasis in the flavinogenic yeast *C. albicans* [60]. In this later species, Sef1 directly binds to the promoter of *RIB1* under iron-limiting conditions [61]. This transcription factor, together with Hap43 and Sfu1 are key regulators in the transcriptional control of iron-responsive genes [61,62]. Under iron-limiting conditions, Hap43 and iron-uptake genes are activated by Sef1 [61]. In contrast, under iron repletion conditions, *SEF1* and iron-uptake genes are repressed by Sfu1 (GATA factor) that, in turn, is repressed by Hap43 under low concentrations of iron [61]. Moreover, Hap43 also regulates the expression of the core HAP complex genes *HAP5*, *HAP32*, and *HAP2*, thus being considered a master regulator of iron homeostasis [62]. Remarkably, Hap43 was found to be involved in the positive regulation of *RIB4* (orf19.410.3) in *C. albicans* under iron depletion conditions [62]. Homologs of those transcription factors were identified in the genome sequence of *C. membranifaciens* IST 626 (Table 3).

Given the taxonomic proximity between *C. albicans* and *C. membranifaciens*, we used the YEASTRACT+ portal [35], a valuable tool that allows cross-species comparative genomics of transcription regulation in nonconventional yeasts [36], to search for putative transcription factor binding sites (TFBS) in the sequences upstream of the genes that code for riboflavin biosynthetic enzymes in *C. membranifaciens*, using as a query the TFBS predicted for TFs from *C. albicans* (Table 2). Remarkably, putative TFBSs for Hap43/Hap5 (CCAAT binding site) or Hap5 (CCATT binding site) were detected in the promoter sequences of several *C. membranifaciens* riboflavin biosynthetic genes and of the candidate genes for the dephosphorylation of ARPP in the riboflavin biosynthetic pathway (Table 2), but no TFBS for Sef1 was identified using this tool. This may indicate that the described TFBS for *C. albicans* Sef1 is distinct to that of *C. membranifaciens*. Nonetheless, the possible regulation of riboflavin biosynthetic genes by Hap43/Hap5 deserves attention and to be experimentally validated. 

Riboflavin transport in and out of the cell is still poorly characterized in flavinogenic yeasts, but two riboflavin permeases and one riboflavin excretase were described in *P. guilliermondii* (reviewed in [63]). More recently, a riboflavin excretase Rfe1 from *C. flareri* was also identified based on homology with *D. hansenii* DEHA2C03784p [64]. Using the same homology approach, we identified a putative riboflavin excretase in the genome sequence of *C. membranifaciens* IST 626 (Table 4). Interestingly, based on the MCL clustering tool [65] at JGI MycoCosm portal, the annotated protein is not conserved among the species considered in the analysis, but is present in the flavinogenic yeasts *C. albicans*, *C. tropicalis*, *M. guilliermondii*, and *D. hansenii*. Concerning the import of the vitamin, in *S. cerevisiae*, an uptake system encoded by *MCH5* that belongs to a family of monocarboxylate transporters was demonstrated to uptake riboflavin into the cell [66]. *C. membranifaciens* IST 626 genome includes a remarkable number of genes encoding proteins from this family, 12, compared with the six identified in *C. albicans* or the five in *S. cerevisiae*. Nevertheless, it is still unknown whether these transporters do have a role in riboflavin uptake in flavinogenic yeasts. The putative TFBS for Hap43/Hap5 were found in the promoter sequences from some of these *C. membranifaciens* encoding genes (Table 4). 

#### 3.4.2. Proteins Associated with Iron Homeostasis and Regulation

The homologs of genes involved in iron homeostasis and regulation, which also include genes encoding iron-dependent flavoproteins, were identified in *C. membranifaciens* IST 626 genome (Table 3). In *S. cerevisiae* and *C. albicans*, the main players of iron metabolism have been identified and characterized (reviewed in [45,67]). In *C. albicans*, this topic has attracted the attention of many researchers due to the extraordinary ability of this species to cope with the different concentrations of iron within the human host microenvironments [68]. In general, extracellular iron uptake in yeasts can occur through the high-affinity reductive iron uptake, which involves extracellular reduction of ferric iron by ferric reductases encoded by the *FRE* genes [69], and subsequent reoxidation to its ferric form by the Fet3 multicopper ferroxidase that makes a complex with the high-affinity iron transporter Ftr1 [70]. Seven ferric reductase encoding genes homologous to *S. cerevisiae* and *C. albicans FRE* genes were identified in the genome of *C. membranifaciens* IST 626, of which six contain an FAD-binding domain. Two *FET3* homologs containing a copper-oxidase domain and one *FTR1* homolog were also identified in the genome sequence of *C. membranifaciens*. In *C. albicans*, five putative multicopper oxidases have been identified, but only four possess the copper-oxidase domains (required for oxidase activity) [71]. Since copper is required for oxidase activity, the intracellular copper transporter Ccc2 is essential for the function of the reductive pathway and for high-affinity iron transport in both *S. cerevisiae* and *C. albicans* [72,73]. We found in the genome of *C. membranifaciens* two putative *CCC2* homologs (Table 3). Remarkably, no *FET4* homolog could be identified in *C. membranifaciens* IST 626 genome. In *S. cerevisiae*, Fet4 is responsible for the low-affinity uptake of ferrous iron [74], and, as found for *C. membranifaciens*, this transporter is absent from *C. albicans* genome [36]. 

The nonreductive iron import machinery in *S. cerevisiae* includes three cell wall mannoproteins (Fit1–Fit3) involved in the retention of siderophore-iron in the cell wall, and four iron-xenosiderophore-specific transporters (Arn1–Arn4). *C. albicans* holds dedicated uptake systems for ferrichrome siderophores (via Sit1/Arn1) and ferrioxamine siderophores (reductive iron uptake—Ftr1-dependent), whereas, in *S. cerevisiae*, Sit1 can facilitate the uptake of ferrioxamines, as well as ferrichrome-type siderophores [75]. In the *C. membranifaciens* genome sequence, as in *C. albicans*, no Fit1–Fit3 homologs were identified, but three Arn1/Sit1 homologs were detected, while *C. albicans* has only one Arn1/Sit1 in its genome (Table 3). 

Yeasts also have the ability to utilize heme or hemoglobin as an iron source, a critical process for yeast survival and virulence previously characterized in *S. cerevisiae* and *C. albicans* [76]. In *C. albicans*, the uptake of hemoglobin is mediated by a family of specific hemoglobin receptors in the cell surface encoded by *RBT5*, *RBT51*, *WAP1/CSA1*, *CSA2*, and *PGA7* genes [77]. In *S. cerevisiae,* this family of hemoglobin transporters is absent, consistent with the, in general, nonvirulent nature of this species. After internalization of hemoglobin inside vacuoles, this molecule is hydrolyzed or denatured to release the heme group that can subsequently be oxidized by the heme oxygenase Hmx1 [76]. As in *C. albicans* and *S. cerevisiae*, *C. membranifaciens* includes in its genome sequence the gene encoding the heme oxygenase Hmx1, as well as the *C. albicans* homologs for hemoglobin receptors Pga10 (Rbt51) and Pga7 (Table 3). 

In *C. albicans*, the activation of the iron regulon by Sef1 is co-ordinated with the biosynthesis of iron–sulfur clusters in the mitochondria [78]. Homologs of the genes involved in iron–sulfur cluster assembly were also identified in *C. membranifaciens* IST 626 genome (Table 3). Iron–sulfur clusters were proposed to play a role in the regulation of riboflavin biosynthesis and iron accumulation in the flavinogenic yeast *M. guilliermondii*, but the mechanisms underlying such an association remain unclear [79].

#### 3.4.3. Proteins Associated with Sugar Transport and Metabolism 

*C. membranifaciens* IST 626 can assimilate a wide variety of carbon sources that include hexoses (glucose and galactose), pentoses (xylose and arabinose), α-glucosides (maltose, trehalose, melezitose), β-glucosides (cellobiose, salicin), β-fructosides (sucrose, inulin), and α-galactosides (raffinose, melibiose), but is unable to assimilate lactose (β-galactoside) (Supplementary file 2). The first steps for the assimilation of sugars involve their cellular uptake or extracellular hydrolysis, followed by uptake of smaller molecules. The genome of *C. membranifaciens* IST 626 holds a total of 45 proteins from the sugar porter family (TC 2.A.1.1) [34,80] (Table 5, Supplementary file 3). This remarkable number of putative sugar transporters includes maltose and general α-glucoside transporters, involved in the transport of trehalose, maltose and/or melezitose, putative glucose/xylose facilitators and proton symporters, hexose transporters, glucose sensors, and transporters for other compounds, such as glycerol or quinate (Table 5, Supplementary file 3). Regardless of *C. membranifaciens’* inability to assimilate lactose, homologs of *K. lactis* galactose/lactose permease Lac12 [81] were found in its genome sequence. It is likely that these putative transporters are responsible for the uptake of galactose instead of lactose. Moreover, no β-galactosidase-encoding gene (KOG0496) was found in the genome of *C. membranifaciens* IST 626 (Supplementary files 1 and 4). 

Regarding the ability of *C. membranifaciens* IST 626 to assimilate sucrose and inulin, two genes encoding invertases (KOG0228) were identified in the genome of this strain (Supplementary files 1 and 4) (Protein IDs 17842 and 18031) and a melibiase-encoding gene was identified as well (Protein ID 18584, KOG2366), confirming this species’ ability to assimilate melibiose (Supplementary files 1 and 4). *C. membranifaciens* is also able to assimilate cellobiose and salicin, and the presence of glycoside-hydrolase-encoding genes (Protein IDs 31127, 19784, 12153; KOG0626) confirmed the suggestion of β-glucosidase activity (Supplementary files 1 and 4). Other enzymes identified in this strain include 3 α-amylases (Protein ID 11615, KOG3625; Protein IDs 18192 and 23493, KOG0471), consistent with its ability to assimilate soluble starch.

## 4. Conclusions

Valuable information regarding the isolation of *C. membranifaciens* strains from soil samples and the general ability of the species to produce riboflavin that, to date, was considered exclusive of *C. membranifaciens* subsp. *flavinogenie* W14-3 was provided. Among the *C. membranifaciens* isolates obtained and tested, strain *C. membranifaciens* IST 626 was selected as the best riboflavin producer and demonstrated that riboflavin production can be improved by culture medium optimization. Medium supplementation with glycine favored complete xylose metabolization, leading to higher biomass concentration and riboflavin volumetric production that reached approximately 120 mg/L after around 200 h of cultivation. *C. membranifaciens* IST 626 genome was sequenced and annotated, providing some indications on riboflavin biosynthesis and regulation and on the assimilation of different carbon sources. Putative transcription factor binding sites for Hap43 transcriptional regulator were found in the promoter regions of riboflavin biosynthetic genes’ homologs (*RIB1*, *RIB3*, *RIB5*, *RIB7*, and *FMN1*), suggesting that Hap43 may have a role in the regulation of riboflavin biosynthesis under iron limitation. In future work, it would be interesting to examine the role of this transcription factor in the regulation of these and other genes, and compare the transcriptional profile of *C. membranifaciens* IST 626 and that of the type strain in different media. *C. membranifaciens* was found to hold a remarkable number of transporters for sugar/sugar-related compounds and metabolic enzymes, consistent with its capacity to use a wide variety of carbon sources, in particular, hexoses, pentoses, and inulin. These carbon sources are present in the hydrolysates from forest and agro-industrial residues, making this species a possible platform for riboflavin production from relevant feedstocks under a circular economy context. In conclusion, this work put forward the riboflavin overproducer *C. membranifaciens* and provides valuable molecular data to be used for the development of novel strains able to effectively use a wide range of raw materials in the production of added-value compounds.

## Figures and Tables

**Table 1:** Genome assembly statistics of *C. membranifaciens* IST 626.

Feature	
Total reads	17,196,026
No. of scaffolds (≥2000 bp)	56
Coverage	224x
N50 (bp)	667,103 bp
L50	7
Maximum contig length (bp)	1,053,043 bp
Minimum contig length (bp)	2057 bp
Assembly size (bp)	11,508,125 bp
GC content (%)	32.15%

**Table 2:** Homologs of flavin biosynthetic genes in *C. membranifaciens* IST 626 and putative transcription factors (TFs) controlling the target gene expression considering the identified transcription factor binding sites (TFBSs) found in the promoter regions of those genes.

Protein ID	Homolog in *C. albicans*	Amino Acid Identity (%)	Molecular Function (*)	Putative TFs Based on Predicted Binding Sites
30281	*RIB1*	74%	GTP cyclohydrolase II	Efg1, Tye7, Brg1, Hmo1, Nrg1, Rap1, Hap43/Hap5, Upc2, Wor3, Mrr1, Ace2, Hap5, Rtg1, Rtg3, Zcf29
15396	*RIB2*	67%	Pseudouridine synthase	Efg1, Tye7, Ndt80, Skn7 (**)
15025	*RIB3*	77%	3,4-dihydroxy-2-butanone-4-phosphate synthase (DHBP synthase)	Efg1, Brg1, Nrg1, Rim101, Hap43/Hap5, Wor3, Mrr1, Hap5, Rgt1, Rtg3, Zcf29, Skn7
34729	*RIB4*	77%	Lumazine synthase (6,7-dimethyl-8-ribityllumazine synthase synthase)	Efg1, Tye7, Ndt80, Nrg1, Upc2, Wor3, Mrr1, Rgt1, Ace2, Zcf29
13130	*RIB5*	69%	Riboflavin synthase	Efg1, Brg1, Nrg1, Rap1, Hap43/Hap5, Mrr1, Rgt1, Ace2, Hap5, Zcf29, Skn7, Cta8
18055	*RIB7*	53%	5-amino-6-(5-phosphoribosylamino)uracil reductase	Brg1, Hap43/Hap5, Mrr1 (**)
25813	*FMN1*	50%	Riboflavin kinase	Upc2, Wor3, Mrr1, Hap5, Zcf29 (**)
24533	*FAD1*	51%	Flavin adenine dinucleotide (FAD) synthetase	Nrg1, Tbf1, Wor3 (**)
34484 (***)	*RHR2*	84%	Predicted haloacid-halidohydrolase	Efg1, Tye7, Ndt80, Brg1, Nrg1, Rap1, Rim101, Hap43/Hap5, Wor1, Mrr1, Mig1, Rgt1, Zcf29, Skn7
23627 (***)	*DOG1*	26%	Predicted haloacid-halidohydrolase	Brg1, Mrr1, Hap5 (**)

(*) Molecular function was retrieved from Candida Genome Database [56]; (**) TFBSs predicted in promoter sequences smaller than 160 bp. (***) Candidate genes for the dephosphorylation of ARPP in riboflavin biosynthetic pathway.

**Table 3:** Homologs of genes involved in iron homeostasis and regulation identified in the genome sequence of *C. membranifaciens* IST 626.

	Protein ID	Homolog in *C. albicans*	Amino Acid Identity (%)	Molecular Function (*)
Regulation of iron metabolism	24521	Sfu1	62%	Zinc finger, GATA-type transcription factor
	23730	Hap43	52%	Basic-leucine zipper domain
	13665	Sef1	67%	Zn(2)-C6 fungal-type DNA-binding domain
	29747	Aft2	41%	Hypothetical transcription factor
	18845	Hap5	79%	Histone-like transcription factor
High-affinity reductive system	16867	Fre10	45%	Ferric reductase (**)
	34201	Cfl2	43%	Ferric reductase (**)
	10230	Fre9	51%	Ferric reductase (**)
	23390	Cfl2	24%	Ferric reductase (**)
	14512	Frp2	39%	Ferric reductase (**)
	31983	orf19.867	26%	Ferric reductase (**)
	20041	orf19.4843	50%	Ferric reductase
	34362	Fet31	69%	Multicopper oxidase
	12903	Fet33	58%	Multicopper oxidase
	29609	Ftr1	74%	High-affinity iron transporter
	35518	Ccc2	49%	Cu2+ transporter P-type ATPase
	34023	Crp1	55%	Cu2+ transporter P-type ATPase
	14754	Atx1	60%	Copper metallochaperone
Siderophore uptake system	14012	Sit1	58%	Siderophore iron transporter
	28384	Sit1	45%	Siderophore iron transporter
	29780	Sit1	26%	Siderophore iron transporter
Heme or hemoglobin utilization system	28257	Hmx1	64%	Heme oxygenase
	25343	Pga10 (Rbt51)	52%	Glycosylphosphatidylinositol (GPI)-modified cell wall protein
	30576	Pga7	57%	Glycosylphosphatidylinositol (GPI)-modified cell wall protein
Iron-sulfur assembly	13591	Isu1	64%	Iron-sulfur cluster assembly
	17237	Nfu2 (orf19.6283)	75%	Iron-sulfur cluster binding activity
	28973	Nfu1 (orf19.2067)	70%	Iron-sulfur cluster assembly
	26437	Isa1	52%	Iron-sulfur cluster assembly
	32180	Isa2	56%	Iron-sulfur cluster assembly
	24028	Dre2	39%	Iron-sulfur cluster assembly
	25680	Yfh1	46%	Mitochondrial matrix protein frataxin, involved in Fe/S protein biosynthesis
	14713	Atm1	72%	mitochondrial ABC transporter; transport of iron-sulfur cluster precursors
	18779	Nar1	54%	Putative cytosolic iron-sulfur (FeS) protein assembly machinery protein
	28349	Nbp35	65%	ATPase activity, iron-sulfur cluster binding activity and role in iron-sulfur cluster assembly
	15021	Nbp35	84%	ATPase activity, iron-sulfur cluster binding activity and role in iron-sulfur cluster assembly
	12204	Nbp35	77%	ATPase activity, iron-sulfur cluster binding activity and role in iron-sulfur cluster assembly

(*) Molecular function retrieved from Candida Genome Database [56]; (**) Contains a FAD-binding domain.

**Table 4:** Candidates for riboflavin transport across *C. membranifaciens* plasma membrane and putative transcription factors controlling the target gene expression considering the identified TFBSs found in the promoter regions of those genes.

Protein ID	Homolog in *C. albicans*	Amino Acid Identity (%)	Molecular Function (*)	Putative TFs Based on Predicted Binding Sites
17681	orf19.3120	56%	Riboflavin excretase. ATP-binding cassette ABC transporter.	Efg1, Tye7, Rap1, Rim101, Cph2, Hap43/Hap5, Mrr1, Ace2, Hap5, Zcf29, Skn7, Cta8, Cph2
5199	orf19.6263	55%	Carbohydrate transport and metabolism|Monocarboxylate transporter	Efg1/Tye7, Ndt80, Wbr3, Mrr1, Rgt1, Hap5, Zcf29, Cta8, Cph2
31581	orf19.6263	34%	Carbohydrate transport and metabolism|Monocarboxylate transporter	Tec1, Efg1/Tye7, Efg1, Brg1, Nrg1, Rap1, Hsm1/Cph2, Hap43/Hap5, Wor3, Mrr1, Mig1, Hap5, Rgt1/Rgt3, Zcf29, Cta8, Cph2
31756	orf19.5720	52%	Carbohydrate transport and metabolism|Monocarboxylate transporter	Nrg1, Wor2, Hap5, Cta8, Cph2
31951	orf19.6263	23%	Carbohydrate transport and metabolism|Monocarboxylate transporter	Efg1, Efg1/Tye7, Rap1, Mrr1, Skn7, Cta8, Cph2
34553	orf19.5720	48%	Carbohydrate transport and metabolism|Monocarboxylate transporter	Tec1, Efg1, Ndt80, Brg1, Rap1, Hap43/Hap5, Wor2, Mrr1, Zcf29, Skn7, Cta8, Cph2
34676	orf19.2751	44%	Carbohydrate transport and metabolism|Monocarboxylate transporter	Efg1/Tye7, Ndt80, Brg1, Rap1, Tfb1, Rim101, Hap43/Hap5, Upc2, Wor1, Mrr1, Zcf29, Skn7, Cta8, Cph2
31444	orf19.6263	53%	Carbohydrate transport and metabolism|Monocarboxylate transporter	Tec1, Efg1/Tye7, Nrg1, Cbf1, Upc2, Wor7, Mrr1, Mig1, Hap5, Skn7, Cta8, Cph2
33236	orf19.4337	45%	Carbohydrate transport and metabolism|Monocarboxylate transporter	Tec1, Brg1, Rap1, Wor2, Mrr1, Hap5, Zcf29, Cta8, Skn7, Cph2
33235	orf19.4337	46%	Carbohydrate transport and metabolism|Monocarboxylate transporter	Brg1, rap1, Rim101, Wor3, Mrr1, Rgt1, Ace2, Hap5, Zcf29, Cta8, Cph2
19539	orf19.4337	27%	Carbohydrate transport and metabolism|Monocarboxylate transporter	Efg1, Efg1/Tye7, Brg1, Nrg1, Rap1, Hsm1/Cph2, Hap43/Hap5, Upc2, Wor3, Wor2, Mrr1, Mig1, Rgt1, Ace2, Hap5, Zcf20, Skn7, Cta8, Cph2
15793	orf19.6209	56%	Carbohydrate transport and metabolism|Monocarboxylate transporter	Mac1, Tec1, Efg1, Efg1/Tye7, Ndt80, Brg1, Rap1, Hap43/Hap5, Wor3, Mrr1, Mig1, Rgt1, Hap5, Zcf29, Skn7, Cta8, Cph2
26147	orf19.6209	56%	Carbohydrate transport and metabolism|Monocarboxylate transporter	Tec1, Efg1/Tye7, Efg1, Ndt80, Brg1, Nrg1, Rap1, Tbf1, Hsm1/cph2, Wor, Mrr1, Rgt1, Hap5, Zcf29, Skn7, Cta8, Cph2

(*) Molecular function retrieved from Candida Genome Database [56].

**Table 5:** Number of sugar transporters from the sugar porter family (TC 2.A.1.1) found in the genome sequence of *C. membranifaciens* IST 626. Transporters are organized into clusters according to the Transporter Classification (TC) system.

Annotation Description (Transporter Classification)	Number of Transporters
Maltotriose/maltose:H+ symporter (2.A.1.1.10)	1
General α-glucoside:H+ symporter (2.A.1.1.11)	7
Glucose/xylose: H+ symporter (2.A.1.1.51)	2
Xylose facilitator (2.A.1.1.40)	3
Glucose/xylose facilitator (2.A.1.1.67)	3
High affinity glucose transporter (2.A.1.1.39)	4
Glucose/Mannose/Galactose/Fructose:H+ symporter (2.A.1.1.43)	2
Lactose/Galactose:H+ symporter (2.A.1.1.9)	6
Glycerol:H+ symporter (2.A.1.1.38)	3
Glycerol uptake permease (Glycerol:H+ symporter) (2.A.1.1.73)	2
Quinate:H+ symporter (2.A.1.1.7)	6
Myoinositol:H+ symporter (2.A.1.1.8)	1
Hexose sensor (2.A.1.1.64)	1
Glucose Transporter/Sensor (2.A.1.1.68)	2
Sugar/Polyol transporter (2.A.1.1.69)	2
Total	45

## Data Availability

The sequences from D1/D2 region of *C. membranifaciens* strains IST 495, IST 498, IST 507, and IST 626 were deposited in GenBank under the accession numbers MZ614941, MW003712, MW003715, and MW532700, respectively. The sequences from ITS region of *C. membranifaciens* strains IST 495, IST 498, IST 507, and IST 626 were deposited under the accession numbers MZ615411, MW003718, MW003721, and MW532702, respectively. The sequencing reads were deposited in Sequence Read Archive (SRA) under the accession number PRJNA777779 and the sequences obtained in the genome assembly were deposited in GenBank under the accession number JAKQXL000000000. The genome annotation is available in JGI fungal genome portal MycoCosm (https://mycocosm.jgi.doe.gov/Canmem1).

## Supplementary Materials

The following supporting information can be downloaded at: https://www.mdpi.com/article/10.3390/jof8030254/s1, Supplementary file 1: Number of predicted genes assigned to a function based on the Eukaryotic orthologous groups of proteins (KOG) classification in *Candida membranifaciens* IST 626, *Candida tenuis* NRRL Y-1498, *Candida tropicalis* MYA3404, *Meyerozyma guilliermondii* ATCC 6260, and *Candida albicans* SC5314. Information was retrieved from MycoCosm portal of Joint Genome Institute; Supplementary file 2: Physiological characteristics of *Candida membranifaciens* IST 626; Supplementary file 3: Putative sugar transporters from the Sugar Porter family (2.A.1.1) found in the genome sequence of *Candida membranifaciens* IST 626. Transporters are organized into clusters according to the Transporter Classification (TC) system; Supplementary file 4: Glycoside hydrolases identified in the genome sequence of *Candida membranifaciens* IST 626 based on the Carbohydrate-Active Enzymes database (CAZy).
